# Toward a better understanding of the ecological roles of extracellular vesicles from plant-associated bacteria

**DOI:** 10.1128/aem.01766-25

**Published:** 2025-11-04

**Authors:** Timothée Zannis-Peyrot, Fanny Nazaret, Céline Lavire, Ludovic Vial

**Affiliations:** 1 Université Claude Bernard Lyon 1, UMR 5557 Ecologie Microbienne, CNRS, INRAE, VetAgro Sup, UCBL, Villeurbanne, Lyon, France; The Pennsylvania State University, University Park, Pennsylvania, USA

**Keywords:** extracellular vesicles, phytobacteria, ecology, multi-omics, phytopathogen

## Abstract

Extracellular vesicles (EVs) from phytobacteria are emerging key ecological actors of plant–bacteria interactions. They can promote colonization of the host by delivering virulence factors and cell wall-degrading enzymes. Extracellular vesicles also modulate the plant immunity by transporting compounds that either induce or suppress plant defense responses. Furthermore, they mediate interspecies and inter-kingdom communication, influencing microbial community dynamics. This review highlights the involvement of bacterial EVs in many parts of the plant–bacteria dialog. We emphasize the need for studying EVs from phytobacteria to better understand plant–bacteria interactions and the possible use of bacterial EVs in a One Health context.

## INTRODUCTION

Plants constantly interact with numerous microorganisms that live on their surfaces (such as roots and leaves) or colonize their tissues, and they interact with them during their entire life cycle (1). These interactions are mediated by the dynamic exchange of various molecules between both the microorganisms themselves and their host, which can lead to numerous effects ranging from beneficial to detrimental for plant health (2, 3). The release of bacterial molecules usually occurs via diffusion through the cell membranes or is mediated by secretion systems, either for secretion into the extracellular environment or for direct injection into the cytoplasm of neighboring cells (4). Bacterial extracellular vesicles (EVs) have gained importance as they have been proposed as an alternative to classical secretion systems (5). These lipidic bio-nanoparticles, with a size ranging from ~50 to 400 nm, allow cells to export a heterologous molecular cargo involved in long-range molecular interactions, thus enhancing the microorganism’s range of action. While their production requires a lot of energy, bacterial EVs can protect their cargo from extracellular threats encountered in intraspecies, interspecies, or inter-kingdom interactions (6–9).

The designation of bacterial EVs, as well as their cargo, depends on the membrane they originate from. In gram-negative bacteria, outer membrane vesicles (OMVs) are the most studied EVs and refer to vesicles emerging from the outer membrane, carrying mostly periplasmic elements. Outer-inner membrane vesicles (OIMVs) possess two layers of membrane resulting from the packaging of an inner membrane vesicle, which carries cytoplasmic elements within an outer membrane layer. Finally, explosive cell lysis induced by prophage-derived endolysins can result in the formation of explosive OMVs (EOMVs) and explosive OIMVs (EOIMVs) (10). In gram-positive bacteria, membrane vesicles refer to monolayer vesicles originating from the cell (11). In this review, we will not discuss gram-positive bacteria EVs, as current knowledge on the subject is scarce and no examples of EV-mediated interactions between gram-positive bacteria and plants have been described yet. The identification of EVs types can be difficult yet essential to understand their cargo and functional effects whether they come from prokaryotes or eukaryotes. The International Society for Extracellular Vesicles issued and updated guidelines named Minimal Information for Studies of Extracellular Vesicles (MISEV) to unify and standardize EVs purification protocols, subtypes identification, and denomination. Following the MISEV 2023 guidelines, we refer to all types of extracellular vesicles described in studies on phytobacteria as EVs (12).

Although their importance is currently less described than in animal–pathogen interactions, bacterial EVs have now been identified as key factors in a growing number of plant–bacteria interactions (Table 1). In particular, numerous studies focusing on the bacterial side have shown that EVs could be involved in the delivery of compounds of interest, such as virulence factors or plant defense elicitors (13). However, while these exciting findings allow for future investigations for a better understanding of molecular determinants in plant–bacteria interactions, the ecological roles of phytobacterial EVs have only been described in a few model systems. Indeed, few studies have investigated both the cargo of EVs and their impact on plants, and current hypotheses are essentially transposed from knowledge accumulated in the context of microbial interactions from other ecosystems (14). In this regard, characterizing the possible ecological roles of EVs from plant-associated microorganisms seems essential to better understand plant–microorganism interactions and their potential use in more environmentally friendly agricultural approaches.

**TABLE 1:** EV studies from plant-associated bacteria^*b*^^,^^*c*^

Relationship with the plant	Bacterial strain	Plant^*a*^	Content analysis	Functional analysis	Ref
Beneficial	*Azospirillum* sp. B510	Tomato	Proteomics, lipidomics, metabolomics, influence of medium (+/− ferulic acid)	Influence on tomato metabolome, immuno-modulation	(15)
	*Pseudomonas fluorescens*	nd	Proteomics, influence of medium (rich vs apolast-mimicking)	nd	(16)
		Arabidopsis (leaves) Tobacco (leaves) Tomato (leaves)	nd	Immuno-induction, protection against pathogens	(17)
	*Pseudomonas chlororaphis* O6	nd	Raman spectroscopy, influence of stresses (CuO and H_2_O_2_)	nd	(18)
	*Rhizobium etli* CE3	nd	Proteomics, influence of growth phase	nd	(19)
		nd	Proteomics, influence of medium (+/− symbiosis mimicking molecule)	nd	(20)
	*Sinorhizobium fredii*	Soybean (roots)	Proteomics, lipidomics, influence of medium (+/− symbiosis mimicking molecule)	Root hair deformation, immuno-suppression	(21)
Commensal	*Pseudomonas putida* KT2440	nd	Proteomics	nd	(22)
		nd	Proteomics, influence of medium (+/− lignin)	*In vitro* catabolism of lignin derivatives	(23)
		nd	Proteomics	Link between T6SS and OMVs cargo sorting	(24)
		nd	Proteomics, lipidomics	nd	(25)
Pathogenic Pathogenic	*Acidorovax citruli*	Arabidopsis	SDS-PAGE	Immuno-activation	(26)
	*Agrobacterium tumefaciens* C58	nd	Proteomics, lipids (2D-TLC)	Role of OM protein in cell and proteoliposomes attachment	(27)
	*Pectobacterium brasilense* 1692	Tobacco	Proteomics	Immuno-activation, antibacterial activity	(28)
	*Pectobacterium zantedeschiae* 9M	Calla lily	Proteomics, influence of medium (+/− potato extract)	*In vitro* pectate degradation, plant tissues maceration, MIC assay	(29)
	*Pectobacterium odoriferum* Car1	Calla lily	nd	*In vitro* pectate degradation, plant tissues maceration, MIC assay	(29)
	*Pseudomonas syringae* DC3000	Arabidopsis	nd	Immuno-activation	(26)
		Arabidopsis (leaves) Tobacco (leaves) Tomato (leaves)	nd	Immuno-activation, protection against pathogens	(17)
		nd	Proteomics, influence of medium (rich vs apoplast-mimicking)	nd	(16)
		Arabidopsis (leaves)	Proteomics	Immunoactivation, protection against pathogen, production of EVs *in planta*	(30)
	*Pseudomonas syringa*e T1	nd	Proteomics, lipids (TLC), LPS	nd	(31)
	*Xanthomonas campestris* pv. *campestris* B100	nd	Proteomics, LPS, influence of medium (rich vs virulence-inducing)	nd	(32)
	*Xanthomonas campestris* pv. *campestris* 33913	Arabidopsis	nd	Immuno-activation, plant RNA-seq analysis	(33)
	*Xanthomonas campestris* pv. *campestris* 8004	Arabidopsis (leaves, roots, protoplasts)	Lipidomics	Immunoactivation, integration of EVs in the plant cell membranes	(34)
	*Xanthomonas campestris* pv. *vesicatoria* 85-10	nd	Immune electron microscopy and Western blot on virulence effectors	nd	(35)
	*Xanthomonas citri* pv. *citri* 306	nd	Proteomics, lipidomics, metabolomics	Contribution of EVs in bacterial nutrition	(36)
	*Xanthomonas oryzae* pv. *oryzae* PXO99	Arabidopsis	Western blot	Immuno-activation	(26)
	*Xylella fastidiosa* spp. *fastidiosa* Temecula 1	nd	Western blot	Production of EVs *in planta,* block cell attachment to xylem cells	(37)
		nd	Proteomics	nd	(38)
		nd	Proteomics, metabolomics	nd	(39)
		Arabidopsis	Proteomics, DNA-seq, RNA-seq, compared to WCL	Downregulation of sRNA gene targets *in planta*	(40)
	*Xylella fastidiosa* spp. *pauca* 9a5c	nd	Proteomics, metabolomics	nd	(39)
	*Xylella fastidiosa* spp. *pauca* De Donno	nd	Proteomics, compared to WCL	nd	(40)
	*Xylella fastidiosa* spp. *pauca* Fb7	nd	Proteomics, metabolomics	nd	(39)

^*a*^ Plant: plant model on which EV effect was tested.

^*b*^ Studies in which EVs from phytobacteria were isolated and studied whether for their cargo content or functions in plant–bacteria interactions.

^*c*^ nd, not determined; LPS, lipopolysaccharides; TLC, thin-layer chromatography; MIC, minimum inhibitory concentration; T6SS, Type VI Secretion System; WCL, whole-cell lysates.

**Fig 1:**
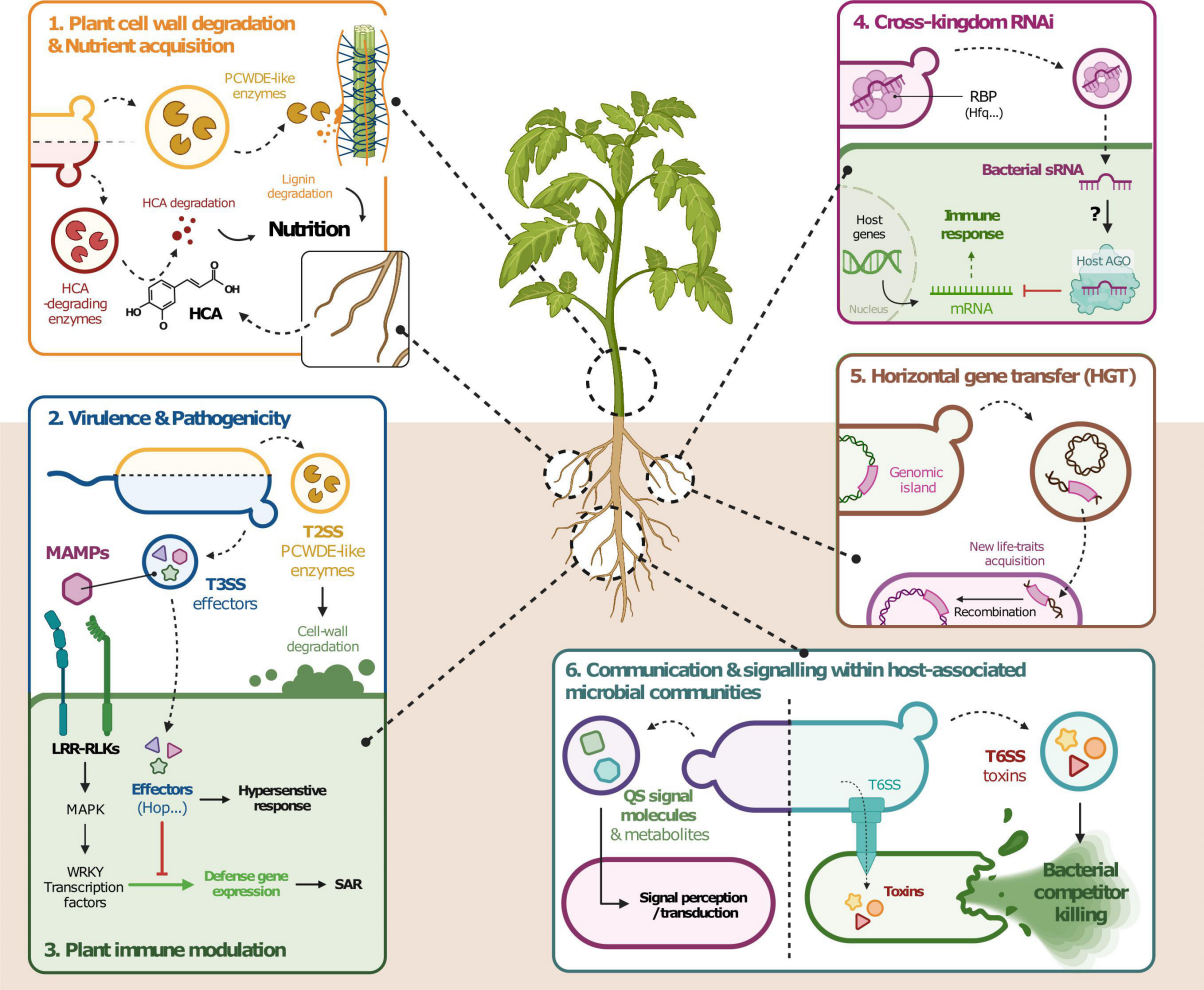
Integrated ecological roles of phytobacterial extracellular vesicles in plant-associated environments. Phytobacteria release extracellular vesicles (EVs) carrying a complex and heterologous molecular cargo, contributing to key ecological processes involved in interspecies, intraspecies, and inter-kingdom interactions at the plant level. These processes include the following. (1) Plant cell wall degradation and nutrient acquisition (top left, orange): EVs may carry PCWDE-like facilitating the degradation of lignin or HCA derivatives, ensuring nutrient acquisition. (2) Virulence and pathogenicity (bottom left, blue): EVs can deliver virulence-associated proteins, such as T2SS effectors and PCWDE-like enzymes, that contribute to cell wall degradation and tissue colonization. (3) Plant immune modulation (bottom left, green): EV cargo might also harbor MAMPs, T3SS effectors, and other virulence factors involved in plant immune modulation. In some documented cases, bacterial EV inoculation leads to induction of MAPK signaling pathway and expression of WRKY transcription factors, leading to SAR establishment. In inoculated cells, some specific effectors and MAMPs carried by EVs can also induce HR. (4) Cross-kingdom RNA interference (RNAi) (top right, pink): EVs can transport regulatory sRNA associated with RBP (i.e. Hfq) that may lead to suppression of host mRNA expression via RNAi pathways involving AGO proteins. (5) Horizontal gene transfer (HGT) (middle right, brown): EVs may mediate the transfer of mobile genetic elements such as GIs from one bacteria cell to another, contributing to the acquisition of new specific traits representing an interest in the plant-environment. (6) Communication within microbial communities (bottom right, light blue): EVs can deliver QS signal molecules and toxins usually transferred via the contact-dependent T6SS. These molecules are generally involved in bacterial communication and can shape microbial community composition in the plant microbiome. PCWDE, plant cell wall-degrading enzymes; HCA, hydroxycinnamic acids; MAMPs, microbe-associated molecular patterns; LRR-RLKs, leucine-rich repeat receptor-like protein kinases; HR, hypersensitive response; SAR, systemic acquired resistance; RNAi, RNA interference; RBP, RNA-binding proteins; sRNA, small RNA; AGO, argonaute protein; HGT, horizontal gene transfer; GIs, genomic islands; QS, quorum sensing; T2SS-T3SS-T6SS: Type 2-3-6 secretion system(s).

This review synthesizes two decades of key advances in the study of EVs from plant-associated bacteria (phytobacteria), linking together the molecular and functional relevance of phytobacterial EVs to plant health from an ecological perspective (Fig. 1), and concluding with future research directions and applied perspectives.

## PLANT CELL WALL DEGRADATION AND NUTRIENT ACQUISITION

Many phytobacteria, especially phytopathogens, colonizing the apoplast or intercellular spaces, release specialized enzymes known as plant cell wall degrading enzymes (PCWDEs) (41). These enzymes are released into the extracellular environment to degrade the plant cell wall barrier either for virulence or nutrition inside the host (42–44). The use of EVs could enhance this process by allowing the delivery of enzyme-enriched cargo upstream of bacterial invasion. Several studies investigating the proteinaceous cargo of phytobacterial EVs have identified enzymes that can remain active independently of the bacteria’s presence. Indeed, EVs from the phytopathogens *Pectobacterium brasiliensis*, *Pectobacterium zantedeschiae*, *Xanthomonas campestris* pv. *vesicatoria*, and *Xylella fastidiosa* carry multiple pectate-degrading enzymes, xylanases, and PCWDE-like enzymes, respectively (28, 29, 35, 39). These enzymes were found to be functional both *in vitro* and *in planta*, with maceration activity against *Solanum tuberosum* tuber tissues for *P. brasiliensis* EVs and necrosis against pepper leaves for *X. campestris* EVs (28, 35).

The plant cell wall can be modified through the addition of lignin (45). *Pseudomonas putida*, a plant growth-promoting bacteria (PGPB), secretes EVs carrying enzymes capable of degrading lignin-derived aromatic compounds (e.g., hydroxycinnamic acids [HCAs]) when this type of molecules is present in its growth environment (23). Interestingly, HCAs and their degradation products are known to play a role in plant defense as they can be harmful to microorganisms (46). Bacteria could use their EVs as tools to detoxify their environment in response to plant defense reaction. HCA-degrading enzymes were also found in the EVs from the PGPB *Azospirillum* sp. B510. As this bacterium is known to induce the production of HCAs by the plant, it could use its EVs in response to the plant metabolic changes for its own nutrition (15, 47).

Additionally, phytobacterial EVs were found to carry multiple substrate-binding receptors. These receptors can be either non-specific, like common sugar receptors, or more specific to plant compounds, such as putrescine-binding receptors (13). Moreover, EVs often harbor many proteins involved in siderophore acquisition (16). In *Xanthomonas citri*, EVs were shown to transport iron, probably through the use of siderophores bound by TonB-dependent receptors (36). Because plant environments, and particularly apoplastic fluids, are relatively poor in bioavailable metals, EVs could extend the reach of essential nutrients during host colonization.

Extracellular vesicles could thus represent a strategy for phytobacteria to acquire resources at great distances from the cell. Two non-exclusive hypotheses have been proposed to support the role of EVs in such nutrient acquisition. The first hypothesis implies that EVs could release their cargo into the surrounding environment, allowing soluble catabolic enzymes to degrade nutrients. The degradation products would then be taken up by the cell directly from the extracellular environment. Alternatively, extracellular substrates could be internalized into EVs to be degraded by catabolic enzymes located within them. Subsequently, the EVs would dock back to the bacterial cell to release their degradation products into the periplasmic space. This hypothesis could provide a clear advantage to microorganisms during nutrient competition if their EVs dock specifically to individuals belonging to their species (23). This idea is reinforced by the fact that Atu8019, a lipoprotein found in EVs from *Agrobacterium fabrum*, can drive the specific docking of proteoliposomes and EVs to itself and phylogenetically close bacteria species (27). However, direct evidence for how such EVs roles are associated with nutrient cellular uptake remains limited and needs to be further investigated.

## VIRULENCE AND PATHOGENICITY

In addition to cell wall-degrading enzymes, the study of EVs from phytopathogenic bacteria revealed the presence of other virulence factors that could help them during host colonization, similar to what has already been described in mammalian pathogens. Indeed, EVs from the human pathogens *Porphyromonas gingivalis*, enterotoxigenic *Escherichia coli*, and *Pseudomonas aeruginosa* have been shown to carry toxins and virulence effectors that can be delivered to mammalian cells (7, 48, 49). In phytobacteria, EVs from *P. syringae* were found to carry virulence effectors from the type III secretion system (T3SS) (16, 28, 30, 31). Notably, they harbor 14 effectors, including HopI1, HopM1, and HopE1, which are known to inhibit plant defense in *Arabidopsis thaliana* by interfering with the cell machinery (50–52).

Interestingly, bacterial EVs seem to serve as an alternative route to traditional secretion systems, leading to their proposition as a type 0 secretion system (5). Indeed, they offer another pathway for the secretion of PCWDEs that are usually secreted by the type II secretion system (T2SS) (38, 53). Additionally, they allow the transport of virulence effectors that are usually delivered directly into eukaryotic host cells by bacterial phytopathogens using the T3SS, as seen in *P. syringae* (16, 54). Moreover, *X. fastidiosa* EVs contain virulence factors, such as LesA lipase, homologous to effectors transported via a T3SS in *X. oryzae*. Since *X. fastidiosa* does not possess a T3SS in its pathogenic arsenal, EVs could be used by the bacteria as a replacement strategy for virulence factors secretion (39, 55).

Additionally, EVs from phytobacteria might contribute to virulence not through their cargo content, but rather by physical interactions between EVs and host cells. Indeed, EVs from *X. fastidiosa* have been shown to reduce bacterial adhesion to xylem vessel walls and thereby facilitate bacterial swarming and movement within the plant (37).

Regarding EVs from plant-associated microorganisms, to date, the majority of studies have focused on the same well-described phytopathogen models (Table 1). However, numerous plant pathogens employ distinct infection strategies involving a wide diversity of virulence effectors. Additionally, many phytobeneficial bacteria also use effectors to colonize the host. Thus, studying a broader range of bacterial models should provide more insights into the roles of EVs during plant colonization and would help identify key virulence factors carried by bacterial EVs.

## PLANT IMMUNE MODULATION

While EVs from plant-associated bacteria may facilitate host colonization through the delivery of functional cargos, they also transport bacterial material that can be detected by the plant, thus modulating its immune response. Specifically, microbe-associated molecular patterns (MAMPs) can be recognized by a diversity of pattern recognition receptors (PRRs) located at the surface of the plant cell membrane (56). In plants, MAMP recognition by PRRs can sometimes lead to the establishment of a hypersensitive response (HR), characterized by the accumulation of reactive oxygen species (ROS) and subsequent necrosis at the infection site due to cell apoptosis. This first line of defense allows the plant to limit the pathogen’s spread through cells (57). For example, EVs from *P. brasiliensis*, *P. syringae*, and *X. campestris* have been shown to transport virulence effectors and to induce HR responses in *Nicotiana benthamiana*, *Solanum lycopersicum*, and *A. thaliana*, respectively (26, 28, 31). Conversely, EVs from numerous phytobacteria transport catalases or superoxide dismutases that could help bacteria overcome HR during plant infection (23, 28–31, 36, 39). This is the case in *P. syringae*, where the catalase KatB was found in EVs and conserved its enzymatic activity. Moreover, both EVs secretion and KatB accumulation increase in the presence of ROS (58).

Beyond inducing local HR, phytobacterial EVs can also act as immuno-activation factors by priming systemic immunity in plants. Indeed, HR is not always triggered at the early stage of a microbial infection, and systemic acquired resistance (SAR) is always induced after the plant’s detection of microorganisms (59). This defense system is regulated by the systemic diffusion of signal molecules in response to the accumulation of salicylic acid (SA) or jasmonic acid (JA) at the infection point (60). These signal molecules stimulate the host defense response by inducing plant cell wall reinforcement and activating metabolic pathways involved in the biosynthesis of secondary metabolites and antimicrobial compounds. SAR allows for extended host protection and is established regardless of the pathogenic nature of the microorganism (61). Acting on plants similarly to the microorganisms from which they originate, bacterial EVs can induce SAR in the plant host. For example, in *A. thaliana*, EVs from the phytopathogens *X. campestris* pv. *campestris*, *X. oryzae* pv. *oryzae*, and *P. syringae* pv. *tomato* induce the expression of SAR-related genes (17, 26). Transcriptome analysis of *A. thaliana* following the inoculation of EVs from *P. syringae* to the roots showed a shift in the immune gene expression profile. Notably, genes encoding WRKY factors, receptor kinases, and receptor-like proteins were upregulated following inoculation, indicating that the plant responded to EVs similarly to its response to a pathogen. Interestingly, 50% of the differentially expressed genes were common with those elicited by the MAMPs flg22, elf18, lipopeptidoglycan, and to a lesser extent, by lipopolysaccharide (LPS) (33). Extracellular vesicles from the phytobeneficial bacterium *P. fluorescens* were also able to induce SAR in *A. thaliana* by inducing the MAPK signaling pathway, rather than the SA signaling pathway induced by EVs from *P. syringae*. This suggests that plants can mount a distinct immune response depending on the ecology of the bacteria producing the EVs (17). Priming roots of *S. lycopersicum* with EVs from *Azospirillum* sp. B510 induced the systemic accumulation of defense metabolites and the modulation of genes involved in their biosynthesis in both roots and shoots. Moreover, this accumulation was shown to be dependent on the EVs’ cargo originating from two different growth environments (15).

Confirming these molecular insights, four different studies demonstrated that priming leaves of *A. thaliana* with EVs from either pathogenic species (*P. syringae, X. campestris*) or beneficial species (*P. fluorescens*) suppressed pathogen infection (17, 30, 33, 34). All these studies compared the effect of EVs on *A. thaliana* to those of purified MAMPs in an attempt to identify a single actor that would explain the action of the EVs. The common conclusion is that immunogenic priming activity cannot be attributed to a single MAMP alone and that the plant response to phytobacterial EVs is complex. Notably, EVs from *X. campestris* were able to induce a plant immune response in a MAMP-independent pathway and may rely on biophysical changes in the plant cell membrane due to the insertion of certain classes of lipids present in *X. campestris* EVs membranes (34). These results offer new perspectives concerning the interaction between bacterial EVs and plants and highlight the need to consider molecular actors beyond just the protein content of EVs.

## CROSS-KINGDOM RNA INTERFERENCE

Bacterial EVs are now known to also carry nucleic acids, including double-stranded DNA, mRNA, and small regulatory RNAs (sRNAs), that can influence host gene expression. Recently, several research studies have particularly focused on characterizing the sRNA content of phytobacteria due to their evident roles in inter-kingdom communication through RNA interference (RNAi) (62). This process can lead to the silencing of specific host genes, particularly those involved in immunity. Such mechanisms are conceptually similar to host-induced gene silencing (HIGS), in which a plant-produced sRNA triggers gene silencing in a target phytopathogen (63, 64). In both cases, the plant RNA silencing machinery, including Dicer-like (DCL) and Argonaute (AGO) proteins, plays a crucial role in processing and guiding the sRNAs to their targets. Until recently, how bacterial sRNAs could be transferred to plant cells remained an open question. Unlike proteins, sRNAs lacked a dedicated export pathway. However, the discovery that EVs contain sRNA cargo and protect them from degradation in the extracellular environment has led to increased interest in their characterization (65, 66). Two recent functional studies characterized the sRNA roles in EVs of phytobacteria, particularly in the modulation of host immune responses (40, 67). On the one hand, RNA-seq analysis allowed the identification of differentially packaged sRNAs in EVs produced by the phytopathogen *X. oryzae* pv. *oryzicola*. Among them, Xosr001 has been found in host plant cells upon EVs inoculation, where it targets *OsJMT1* transcripts, thereby reducing methyl jasmonate (MeJA) accumulation and attenuating JA-mediated defense responses in rice leaves (67). On the other hand, *X. fastidiosa* produces EVs containing nucleic acid involved in gene expression regulation in bacterial and plant cells (regarding RNA cargo) and possibly in horizontal gene transfer (HGT) of genomic islands (GIs) (40). Interestingly, the targets of some sRNAs detected in *X. fastidiosa* EVs are downregulated after inoculation with purified EVs, supporting their involvement in cross-kingdom RNAi. Moreover, proteomic analyses have allowed the identification of RNA-binding proteins (RBPs) enriched in the bacterial EVs, including the RNA chaperone Hfq (40). This is consistent with the fact that many sRNAs require Hfq or other RBPs for stability and target recognition (68, 69). Altogether, these perspectives highlight the need for a deeper functional and mechanistic understanding of the sRNA content in phytobacterial EVs.

## COMMUNICATION WITHIN MICROBIAL COMMUNITIES AND HORIZONTAL GENE TRANSFER

In the plant environment, phytobacteria interact with multiple other microbes within different plant compartments (i.e., the phyllosphere and rhizosphere) through intra- or interspecies exchanges. These interactions can have indirect beneficial effects, such as the suppression of a pathogen by beneficial bacteria, or detrimental effects by coordinating pathogen infection (1). Bacterial coordination plays an important part in host colonization, and many bacteria use quorum-sensing (QS) communication to regulate their colonization processes (70). Quorum-sensing signal molecules belonging to the Diffusible Signal Factor (DSF) family were found in *X. fastidiosa* EVs and were involved in their production. This transport could help the diffusion of such apolar molecules in the bacterial environment toward recipient cells, thus benefiting the bacteria during both plant and insect colonization (39). Bacterial EVs could also help bacteria to ensure the establishment of ecological niches by delivering antimicrobial compounds to co-existing threats. For example, EVs from *Rhizobium etli* were shown to have antimicrobial activity against *Bacillus subtilis*, probably due to the activity of peptidoglycan-modifying enzymes (19). Additionally, EVs from *P. brasiliensis* were shown to transport type VI secretion system (T6SS) toxins that inhibit the growth of *Dickeya dadantii in vitro*. As with the T2SS and T3SS, bacterial EVs seem to serve as an alternative to the T6SS, which could allow for long-distance bacterial warfare. Extracellular vesicles from phytobacteria could also play a role in bacterial defense against fungi that produce antibiotics in the plant environment. Indeed, EVs from *Pectobacterium* spp. carrying β-lactamases were shown to protect sensitive *E. coli* against ampicillin (29). This might be a general mechanism, as it was also found in other well-known bacterial models like *Staphylococcus aureus* and *E. coli* (71, 72).

Within microbial communities, intra- and interspecies communication is not limited to the exchange of chemical compounds. In the past few years, it has been shown that EVs can also act as shuttles for the transfer of genetic material, delivering “DNA-encoded messages” that may modulate community dynamics. Nucleic acid transfer is indeed a key driver of bacterial evolution, thus representing a real source of genetic diversity and plasticity. Until this point, the major mechanisms of HGT between bacteria were limited to natural transformation, conjugation, and transduction (73). The existence of a fourth HGT mechanism mediated by EVs, termed “vesiduction,” has been revealed by several studies (74). The transfer of bacterial genetic material is particularly relevant in soil ecosystems and within plant-associated microbial communities. In the rhizosphere, high microbial densities, close cell proximity on root-associated biofilms, and important carbon fluxes from root exudates facilitate gene exchange (75, 76). In such environments, HGT has been shown to influence plant health by strongly driving the exchange of bacterial traits in both pathogens and beneficial bacteria (77, 78). However, DNA is presumed to be short-lived due to the rapid enzymatic degradation by DNase. The association of DNA with EVs, whether internally or externally, could therefore significantly enhance DNA stability, thus increasing the persistence and transferability of genetic material. Consequently, exploring DNA in phytobacterial EVs and vesiduction would be essential to better understand genetic diversity and plasticity in plant-associated microbial communities, which might considerably influence plant health and both microbial community composition and functions. This perspective is particularly relevant in light of a recent study on *X. fastidiosa*, which revealed the presence of DNA-binding proteins (DBP) and DNA associated with EVs surface, whose sequences are enriched in GIs likely derived from previous HGT (40).

Finally, from an inter-kingdom communication perspective, EVs-mediated transfer of genetic material from bacteria to plants represents an unexplored but very intriguing question. As previously outlined, *A. fabrum*, the inter-kingdom gene transfer of bacterial T-DNA to plant cells, plays a key role in the induction of plant disease (79). Although T-DNA transfer via type IV secretion system (T4SS) is well characterized, the potential for EVs to transfer T-DNA to host plants under specific ecological conditions remains to be elucidated.

## TO AN INTEGRATIVE ANALYSIS OF BACTERIAL EVs COMPOSITION

The cargo of phytobacterial EVs is a key vector of intercellular interactions, both for inter-kingdom exchanges with the plant and interspecies communication within microbial communities. Most studies have focused on EVs content extracted from generic lab media. However, the plant environment has been shown to modulate the cargo of EVs in many phytobacteria (15, 16, 20, 21, 23, 29). The cargos of EVs from phytobacteria grown in a plant-mimicking environment are likely to be enriched in molecular compounds (PCDWEs, MAMPs) that facilitate plant colonization. These results indicate that *in vitro* studies using generic lab medium for bacterial growth and EVs extraction might generate a cargo that does not reflect *in planta* conditions. Extracting EVs from plant-mimicking growth medium (i.e., specific plant molecules, plant exudates) or even from the plant environment (plant fluids, rhizosphere) would help to better appreciate the roles of EVs in phytobacteria ecology.

Thus far, most studies on phytobacteria EVs relied on proteomic analysis to study their cargo and explain their effect on plants. While this method is highly relevant and increasingly sensitive, as many known MAMPs are proteins, it may limit our overall comprehension of the roles of EVs in plant–bacteria interactions. As we discussed earlier, lipidomic analysis is another promising technique as lipids and their derivatives are the common components of EVs. Indeed, EVs formation drives selective enrichment of specific lipids involved in membrane curvature, thereby modifying the lipid relative abundance compared to the cells from which they originate (80). This difference in lipid composition has been shown only in three phytobacterial models so far: *A. fabrum*, *Pseudomonas chlororaphis*, and *X. citri* (18, 27, 36). Moreover, it has been demonstrated that fusion of bacterial EVs to host cell is dependent on both the lipidic composition of EVs and the cell membrane, highlighting the importance of lipids in EV-mediated interactions (34, 81). Additionally to lipids, LPS is found at the surface of EVs from gram-negative bacteria and is implicated in both bacterial virulence and host immunity modulation (82). Interestingly, since LPS tuning can be used by bacteria to evade host detection, EVs harboring such LPS could act as decoys, allowing the bacteria to lure the plant prior to cell infection. It could be the case in phytobacteria where EVs from *P. chlororaphis*, *X. campestris*, and *P. syringae* exhibit distinct LPS profiles compared to the bacterial cell (18, 31, 32). Moreover, LPS influences the surface charge and hydrophobicity of EVs, thus influencing their potential fusion to recipient cells (83, 84).

Bacterial EVs can also transport molecules that cannot diffuse freely through bacterial membranes. For example, *Paracoccus* sp. use their EVs to transport long-chain *N*-acyl-homoserine lactones involved in QS-dependent communication (85). In phytobacteria, EVs from *X. fastidiosa* were shown to transport long-chain molecules from the DSF family involved in the plant colonization by the pathogen (39). This study and two others used untargeted metabolomic analyses on phytobacterial EVs to show the presence of diverse metabolites in their cargos (i.e., amino acids, fatty acids, phenolic compounds, phytotoxins) (15, 31, 39). Given the importance of metabolite compounds in plant–bacteria interaction, it would be interesting for future studies on EVs from phytobacteria to pay due attention to this aspect of their cargos.

It is also tempting to speculate that phytobacterial EV-associated DNA and/or sRNAs also mediate communication with the plant host or directly inside plant-associated microbial communities. Yet, the overall process of cross-kingdom RNAi mediated by EVs remains poorly explored, and its prevalence, functional relevance, and mechanistic basis across phytobacterial species are still largely undescribed. A critical next step will be to determine whether specific sRNAs are selectively enriched in EVs compared to the cellular compartment, and whether subpopulations of EVs carrying distinct regulatory sRNAs exist. These emerging questions could be addressed with single-vesicle RNA-seq technologies, which have been recently adapted from single-cell transcriptomics (86, 87). Combined with flow cytometry analyses, such approaches would enable high-resolution mapping of sRNA content at the single EV level (88).

**Fig 2:**
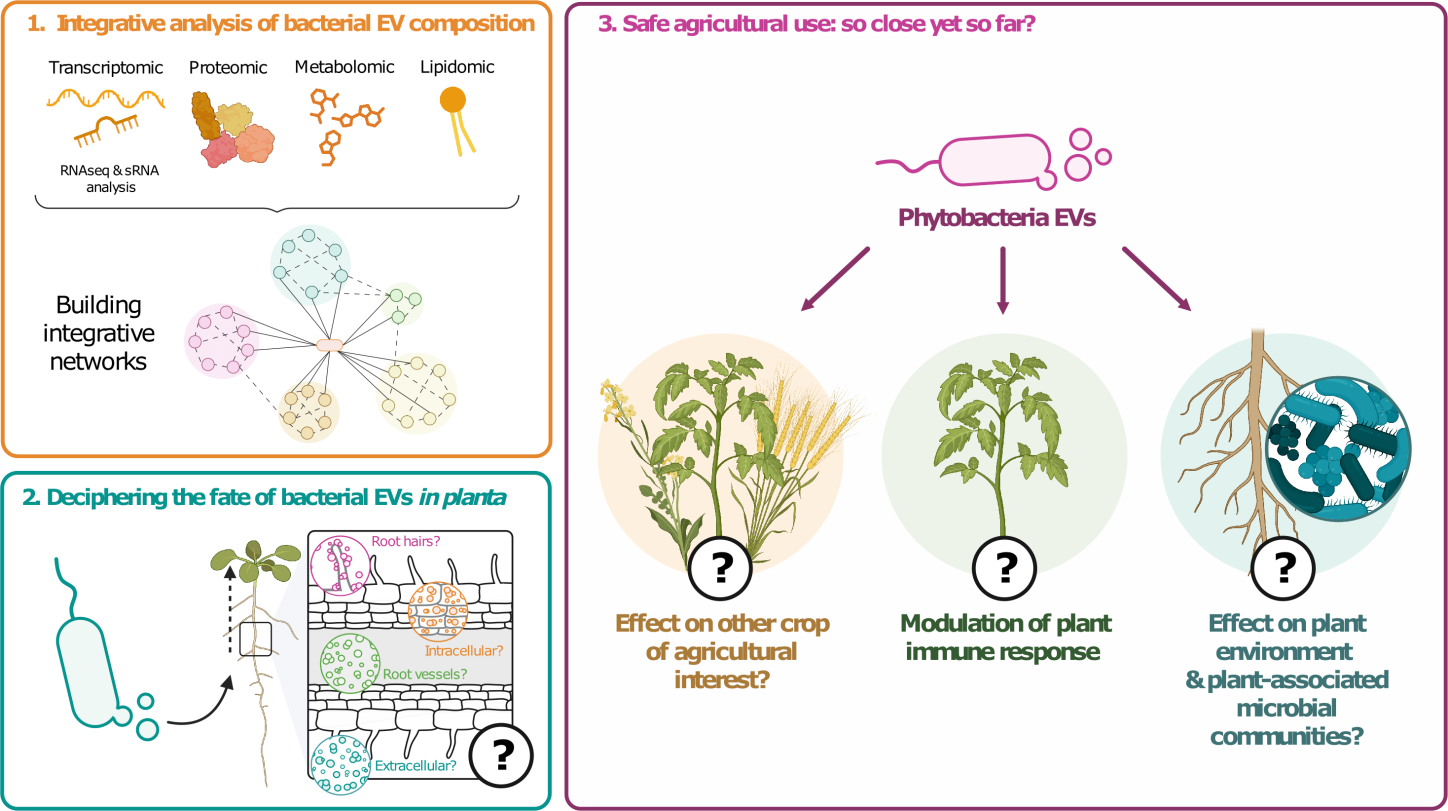
Proposed avenues for future studies focusing on EVs from phytobacteria. So far, research on phytobacterial EVs has brought a lot of exciting results that have raised many more questions regarding their ecological roles. We propose these non-exhaustive directions to better understand these key actors in plant–bacteria interactions. (1) Integrative analysis of bacterial EVs composition (top left, orange): multi-omics studies would help identify key molecules to help understand the roles of EVs in phytobacteria ecology. (2) Deciphering the fate of bacterial EVs *in planta* (bottom left, blue): studying the fate of bacterial EVs *in planta*, especially their targets, potential fusion, and cargo delivery to plant cells, would bring new insights on how they can help bacteria in the plant environment. (3) Safe agricultural use: so close yet so far (left, purple): while EVs represent an exciting tool in a One Health context, multiple questions need to be addressed. Can the actual research on a few plant models be transposed to crops of agricultural interest? Would the immune response modulation seen *in vivo* be enough to protect plants in a field context? Are bacterial EVs safe to use, or could they be detrimental to the plant environment and plant-associated microbial communities?

As we discussed through the manuscript, every part of the molecular cargo of bacterial EVs seems to be relevant when studying plant–bacteria interactions mediated by bacterial EVs. Future studies should therefore not be limited to a single type of cargo component but rather use integrative multi-omics approaches (89). Indeed, these approaches can help shed light on links between different cargo components and lead to a more comprehensive understanding of EVs as a whole and their potential ecological roles in plant–bacteria interactions (Fig. 2).

## DECIPHERING THE FATE OF BACTERIAL EVs *IN PLANTA*

While bacterial EVs production in controlled laboratory environments is clear, studying bacterial EVs production and fate *in vivo* remains challenging. The host environment was shown to influence the EVs production in *V. cholerae,* where the bacteria hypervesiculate to quickly adapt to the host environment upon infection (90). Does this also occur for phytobacteria during plant colonization? *P. syringae* was shown to produce EVs *in planta* using scanning electron microscopy and nanoparticle tracking analysis (NTA). Particularly, zeta potential measurement was used to distinguish bacterial and plant EVs in recovered apoplastic fluids (30). In grape plants, the presence of EVs from *X. fastidiosa* was assessed by combining deconvolution microscopy and FM4-64 labeling, NTA, and marker protein XadA1 abundance in xylem sap fluids recovered from infected plants (37). These two studies showed that EVs produced by the bacteria during colonization were influenced *in planta*. Once produced, bacterial EVs need to navigate to reach the host or other microbial cells. In animal-bacteria interaction, EVs from gut microbiota have been shown to be able to cross the intestinal epithelial barrier by different pathways, either paracellular or intracellular, and enter the bloodstream (91). In plants, one of the major questions regarding EVs, either from plant or microbial origin, is their ability to navigate through plant vessels. More importantly, deciphering if and how bacterial EVs can cross the plant cell wall and their possible uptake by plant cells should be a priority research field (Fig. 2).

## SAFE AGRICULTURAL USE: SO CLOSE YET SO FAR?

To date, studies on phytobacterial EVs have been conducted on simplified systems that do not necessarily reflect a real ecological system such as the rhizosphere. As in any new field of research, it was necessary to understand simple interactions in order to grasp the importance of EVs in plant–bacteria interactions. However, future studies on the roles of phytobacterial EVs in a complex plant environment like the rhizosphere might benefit from the intensive research ongoing in the medicine field, especially on the role of EVs in the human gut microbiome (92).

Based on all these promising results, EVs from phytobacteria have been proposed as new ecological agents for biological control and phytostimulation (93). Indeed, their ability to stimulate plant defenses, combined with their potential antimicrobial activity while being natural and replication-free nature, makes them suitable tools in sustainable agriculture. Yet, many points need to be addressed in order to reach this goal. What is the stability of bacterial EVs? In order to be used as alternatives to classic pesticides, the stability of these nanocarriers needs to be assessed. To date, no study has investigated the impact of storage or buffer on phytobacterial EVs, and all the current knowledge on EVs storage comes from mammalian EVs (94). For how long and against what range of pathogens can bacterial EVs activate the immune defense system? As for now, most of our knowledge is centered around the protection of *A. thaliana* against a few pathogenic models, such as *P. syringae*. The ability of bacterial EVs to protect the plant should be tested against a broader range of phytopathogens, including fungi. How long can this protective effect last? Future studies should investigate the longevity of protective effects of bacterial EVs on different crops of agronomical interest. Finally, how do bacterial EVs affect the plant environment? As discussed above, bacterial EVs can mediate the transport of many compounds such as toxins, antibiotic degradation enzymes, or sRNA. As such, their impact on the environment, from commensal microbial communities to the whole plant ecosystem, should be investigated to ensure their safe use in a One Health context (Fig. 2).
